# Bacterial communities associated with wood rot fungi that use distinct decomposition mechanisms

**DOI:** 10.1038/s43705-022-00108-5

**Published:** 2022-03-30

**Authors:** Irshad Ul Haq, Benjamin Hillmann, Molly Moran, Samuel Willard, Dan Knights, Kathryn R. Fixen, Jonathan S. Schilling

**Affiliations:** 1 Department of Plant and Microbial Biology, College of Biological Sciences, University of Minnesota, St. Paul, MN USA; 2 Biotechnology Institute, College of Biological Sciences, University of Minnesota, Minneapolis, MN USA; 3 Department of Computer Science and Engineering, University of Minnesota, Minneapolis, MN USA; 4 Department of Life Sciences, Imperial College London, London, UK

**Keywords:** Microbiome, Microbial ecology

## Abstract

Wood decomposer fungi are grouped by how they extract sugars from lignocellulose. Brown rot fungi selectively degrade cellulose and hemicellulose, leaving lignin intact, and white rot fungi degrade all components. Many trees are susceptible to both rot types, giving carbon in Earth’s woody biomass, specifically lignin, a flexible fate that is affected not only by the fungal decomposition mechanism but also the associated microbial community. However, little is understood about how rot type may influence the microbial community in decaying wood. In this study, we quantified bacterial communities associated with *Fomes fomentarius* (white rot) and *Fomitopsis betulina* (brown rot) found on a shared tree host species, birch (*Betula papyrifera*). We collected 25 wood samples beneath sporocarps  of *F. fomentarius* (*n* = 13) and *F. betulina* (*n* = 12) on standing dead trees, and coupled microbial DNA sequencing with chemical signatures of rot type (pH and lignin removal). We found that bacterial communities for both fungi were dominated by *Proteobacteria*, a commonly reported association. However, rot type exerted significant influence on less abundant taxa in ways that align logically with fungal traits. Amplicon sequence variants (ASVs) were enriched in *Firmicutes* in white-rotted wood, and were enriched in *Alphaproteobacteria*, *Actinobacteria* and *Acidobacteria* in lower pH brown rot. Our results suggest that wood decomposer strategies may exert significant selection effects on bacteria, or vice versa, among less-abundant taxa that have been overlooked when using abundance as the only measure of influence.

## Introduction

Fungi in natural environments play an important role in carbon cycling due to their ability to decompose wood and other organic material [1, 2] using distinct decomposition mechanisms. White rot fungi decompose lignin as well as cellulose and hemicellulose in the wood cell walls of a wide variety of angiosperms and gymnosperms [3]. These fungi harbor genes encoding ligninolytic enzymes including manganese and lignin peroxidases that are involved in the direct or indirect oxidation of lignin resulting in a stringy or bleached appearance of wood during advanced decay stages and release of cellulose and hemicellulose [4]. Brown rot fungi selectively metabolize cellulose and hemicellulose via extensive depolymerization, leaving lignin partially intact [4–6] and, at least theoretically, “steering” more carbon to soils in lignin residues than to the atmosphere as CO_2_. Given that most of Earth’s aboveground biomass carbon is in wood [7] and that the fate of carbon in wood is flexible depending on ecological factors [8, 9], understanding relationships between rot type and other microorganisms, notable bacteria, is of logical benefit for predicting carbon release from wood.

Bacterial relationships with fungi can range from being commensal or mutualistic to being competitive or antagonistic [10–13], and these types of interactions are likely to influence the deadwood environment. Although little is understood about the types of bacterial–fungal interactions found in deadwood, bacterial community composition in deadwood has been shown to be affected by several factors including decay stage [14] tree species [15], and debris types in temperate forests [16]. However, very little is known about how different decay mechanisms influence bacterial community composition, even though distinct mechanisms of decomposition can alter the availability of many metabolites in decomposing wood microenvironments. The white rot fungus *Fomes fomentarius* (Order: Polyporales) and the brown rot fungus *Fomitopsis betulina* (Order: Polyporales) are efficient wood decomposers but with different preferences for the decomposition of lignin and holocellulose, which includes cellulose and hemicellulose [17, 18]. When compared on the same substrate, birch, *F. fomentarius* was shown to be the quickest decomposer accounting for a 66.84% lignin loss with relatively little decomposition of the holocellulose [19], while *F. betulina* preferentially decomposed the holocellulose resulting in 73.63% mass loss [19]. These different mechanisms of decay resulted in the release of metabolites and carbon substrates from the same substrate (birch) that are quantitatively as well as qualitatively variable [19]. Given this, it seems likely that the release of different metabolites by different wood rot mechanisms influences the surrounding microbial community in these niches and could potentially influence bacterial associations with wood rot fungi.

To determine if differences in the composition of the bacterial communities correlate with distinct mechanisms used by a white rot fungus or a brown rot fungus when decaying the same substrate, we collected samples from standing birch trees with fungal sporocarps of both the white rot fungus *F. fomentarius* and the brown rot fungus *F. betulina*. Samples were collected by drilling into wood at the base (zone of bark penetration for emergence) of each respective fungal sporocarps, and a total of 25 samples (*F. fomentarius* *n* = 13; *F. betulina* *n* = 12) were collected. After verifying wood dominance of either *F. fomentarius* or *F. betulina* using both ITS amplicon sequencing and wood lignin and pH analyses, we used 16S rRNA gene amplicon sequencing to determine the composition of the bacterial communities found in each sample. We show that distinct decomposition mechanisms employed by *F. fomentarius* and *F. betulina* in deadwood are associated with bacterial communities that are distinct in their composition.

## Materials and methods

### Study sites and sample collection

The study site was located at the Cloquet Forestry Center Forest, University of Minnesota, USA (46.7003772475594, −92.54874256381254). Wood drill shaving samples were collected into sterile 50 mL vials by drilling dead standing birch trees (*Betula papyrifera*, Betulaceae, Angiosperms). Samples were first caught in autoclave-sterilized foil used to wrap drill bits (0.5 diameter), and then funneled into sterile 50 mL vials. Most, but not all trees sampled had both *F. fomentarius* and *F. betulina* sporocarps emerging at different locations on the same tree (Supplementary Table 1). Upon removal of sporocarps (for both fungi easily identifiable to species), autoclave-sterilized drill bits (0.5 diameter) were used to extract wood at a depth of 10 cm starting from the location of fungal sporocarps emergence through the bark, drilling perpendicular to the wood grain, and aiming for the pith. In total, 25 samples were collected from standing dead trees in December 2018 and January 2019 at below-freezing temperatures. All samples were frozen and shipped to the laboratory for further processing.

### pH and lignin measurements of drilled wood samples

For each drilled wood sample, 50–75 mg of sawdust was taken in 1.7 mL microcentrifuge. Depending on the sample availability, the exact amount of CaCl_2_ solution (prepared from anhydrous CaCl_2_) needed for each sample was first calculated and then added to the respective tubes (0.1 mL of 5 mM CaCl_2_ per 1 mg of wood). The tubes were incubated at room temperature for 1 h before measuring pH using a calibrated pH probe (Acumet Basic AB15, Fisher Scientific).

For measurement of wood lignin, samples were first dried at 100 °C for 24 h and 60 mg of the oven-dried samples were used for analyses. Acid-insoluble lignin (Klason lignin) was measured gravimetrically after using 0.6 mL of 72% H_2_SO_4_ to digest the material, as described in [20]. Because brown rot fungi remove little lignin, relative to white rot fungi, we used a well-established threshold [20] to delineate brown (<0.8) from white rot (>0.8) for the ratio of lignin loss to density loss (L:D). In our case, without density measurable in drilled samples, we could not determine the extent of decomposition via density loss, other than knowing the wood remained sound enough to drill and contained sapwood (below decay class 4 in 5-class system of [21]). Therefore, we set a very conservative lignin content value of 30.2% for non-adjusted (without adjusting to compensate for density loss) lignin content, using a mean 0.17 g cm^−3^ density for decay class 4 *Betula papyrifera* [22], that would yield a 0.8 L:D threshold. If lignin concentrations, without adjusting to compensate for density loss, exceeded 30.2%, birch decay is predominantly brown rot. In our case, brown rot dominated in majority of the *F. betulina* samples, and in no cases with *F. fomentarius*, implying that this approach was very robust.

### DNA extraction and 16S rRNA gene amplicon sequencing

The DNA was extracted from 25 mg (amount recommended by Qiagen) of homogenized sawdust using the Qiagen’s PowerSoil Pro kit according to the manufacturer’s protocol. For running quality control we used following internal transcribed spacer (ITS) primer pairs: (ITS1) ITS1f: CTTGGTCATTTAGAGGAAGTAA [23]; (ITS1) ITS2r: GCTGCGTTCTTCATCGATGC [24]; (ITS2) 5.8SR:TCGATGAAGAACGCAGCG [25, 26]; (ITS2) ITS4:TCCTCCGCTTATTGATATGC [24] for Basidiomycetes to verify wood decay dominance by each fungus targeted. As described in [27], the V4 hypervariable region of the bacterial 16S rRNA gene was amplified using 515F and 806R primer pair (Meta_V4_515F: TCGTCGGCAGCGTCAGATGTGTATAAGAGACAG**GTGCCAGCMGCCGCGGTAA**; Meta_V4_806R: GTCTCGTGGGCTCGGAGATGTGTATAAGAGACAG**GGACTACHVGGGTWTCTAAT**) [28]. The indexing primers used were: forward indexing primer **AATGATACGGCGACCACCGA**GATCTACAC[i5]TCGTCGGCAGCGTC; reverse indexing primer: **CAAGCAGAAGACGGCATACGA**GAT[i7]GTCTCGTGGGCTCGG. KAPA HiFidelity Hot Start Polymerase (Roche) was used to perform all polymerase chain reactions (PCRs). Prior to the two PCRs (i.e., PCR 1 and PCR 2), an initial amplification of the V4 region was done using qPCR to quantify 16S rRNA gene copy number. The samples were then normalized based on molecule number prior to library preparation (sample input was normalized based on molecule number vs. dsDNA concentration). For qPCR the following conditions were used: 95 °C for 5 min, 35 cycles of 98 °C for 20 s, 55 °C for 15 s, and 72 °C for 1 min, followed by 5 min at 72 °C. All samples were normalized to 1.67 × 10^5^ molecules µL^−1^ after the completion of qPCR based on the volume (3 µL) of sample used for PCR 1, so 5 × 10^5^ molecules is roughly 10× the target sequencing coverage. For PCR 1 and PCR 2 the cycling parameters were like that of the qPCR except the number of cycles which were 25 and 10 for PCR1 and PCR2, respectively. The resulting amplification products from PCR 1 were diluted (1:100) and 5 µL of the diluted product was used in PCR 2. For qPCR and PCR1 Meta_V4_515F/Meta_V4_806R primer pair was used whereas PCR2 was performed with forward and reverse indexing primers. Samples were pooled and denatured with NaOH (1 mM). Before loading, samples were diluted to 8 pM in HT1 buffer (Illumina), then spiked with 15% PhiX, and heat denatured at 96 °C for 2 min. Two blanks (water; no nucleic acid) were included and eluted with Qiagen Solution C6 (10 mM Tris-HCl, pH 8.5). For sequencing, MiSeq 600 cycle v3 kit was used generating 300 bp paired-end reads. DNA extractions, dual-index microbiome amplifications and sequencing of all samples was carried out at the University of Minnesota Genomics Center (Minneapolis, Minnesota, USA).

### Sequence data processing

Raw Illumina sequencing reads were analyzed using the self-learning quality control pipeline SHI7 v0.9.9 [29] to remove sequencing adapters, ascertain reads stitchablity and accomplish quality control of the sequencing data. The parameters used can be found on https://github.com/IrshadUlHaq1/parameters.

### Bioinformatics and statistical analyses

The resulting amplicon sequences were imported to QIIME2 (version 2021.4) [30] using QIIME2 import plugin. After importing to QIIME2 with the manifest file, the sequences were demultiplexed using the q2-dmux plugin. The demultiplexed sequences were then denoised and quality filtered using the Divisive Amplicon Denoising Algorithm (DADA2) [31] by running the q2-dada2 plugin, and three output files including ASV table, representative sequences and denoising statistics were generated. The ASV table and representative sequences were explored by generating visual summaries using the QIIME2 “feature table summarize” and “tabulate sequence” plugins, respectively. Next, we built a fragment-insertion-based phylogenetic tree by employing SATé-enabled phylogenetic placement (SEPP) technique using the q2-fragment-insertion plugin [32] with “sepp-refs-silva-128.qza”. Using q2-fragment insertion, we filtered our feature table to retain fragments that are present in the insertion tree and discard SEPP-rejected fragments. The taxonomic classification of the reads was performed with q2-feature-classifier plugin using a pretrained classifier (Silva-138-99-515-806-nb-classifier.qza) that is based on naïve Bayesian algorithm [33–35]. For the training of the classifier scikit-learn 0.23.1 was used [36]. Taxonomy plots were generated by employing the q2-taxa barplot plugin. Visualization of the taxa barplot allowed us to look at the ASVs associated with all samples and the two blank controls. For the identification of contaminant taxa in our dataset, we used prevalence model of decontam v1.8.0 [37] with default classification threshold (P* = 0.1) as well as a more stringent threshold (P* = 0.5). One ASV (Feature ID: 5ad0bdb355eee69fed1de03984625e) corresponding to *Pseudomonas* was flagged as potential contaminant in our samples, which was filtered out using q2-feature table plugin. All ASVs representing chloroplasts and mitochondria were removed from the dataset using the q2-taxa plugin. Based on a minimum relative abundance threshold (20%) of *F. fomentarius* and *F. betulina* (Supplementary Fig. 1), 17 out of 25 samples were retained while 8 samples with relative abundances less than the 20% threshold were filtered out. Rarefaction plots were generated using three depths (1189, 1782, and 2653) with q2-diversity alpha rarefaction plugin. Samples were rarefied to a depth of 1189 sequences per sample. We chose this depth to retain maximum number of samples and maintain enough statistical power for downstream analyses. Next, we performed diversity analyses applying core-metrics-phylogenetic method with random sampling to a depth of 1189 using q2-diversity plugin. Alpha diversity metrics included Shannon entropy, Pielou’s evenness, observed features and Faith’s Phylogenetic Diversity (Faith PD) [38], whereas beta diversity metrics comprised of weighted UniFrac [39], unweighted UniFrac [40], Bray-Curtis dissimilarity [41, 42] and Jaccard distances [43]. The diversity analyses also generated Principal Coordinate Analysis (PCoA) plots for the above-mentioned beta diversity metrics using q2-emperor plugin.

We then performed statistical tests (Kruskal-Wallis test—nonparametric statistical test) on all metrics of alpha diversity using our categorical metadata, with the alpha-group-significance method of the q2-diversity plugin. Similarly, for all beta diversity metrics, the beta-group-significance method in the q2-diversity plugin was used with pairwise Permutational Multivariate Analysis of Variance (PERMANOVA) to test if bacterial communities associated with *F. fomentarius* were statistically different from *F. betulina*-associated communities in beta diversity. The differences in relative abundances of the four dominant phyla (*Proteobacteria*, *Firmicutes*, *Actinobacteria,* and *Acidobacteria*) in *F. fomentarius* and *F. betulina* samples were tested for significance using Kruskal-Wallis test in RStudio (version 2021.09.1 + 372). Differentially abundant ASVs between the two fungal species (*F. betulina* vs *F. fomentarius*) were calculated by using the analysis of compositions of microbiomes with bias correction (ANCOM-BC) method [44] in RStudio (version 2021.09.1 + 372), with parameters listed here: https://github.com/IrshadUlHaq1/parameters.

The statistical analyses of the pH (as converted H ion concentrations) and lignin data were performed in RStudio (version 2021.09.1 + 372). Using the Shapiro test on individual populations (*F. betulina* and *F. fomentarius*), data was confirmed to be normally distributed. Statistical significance was determined with independent *t*-test.

For data visualization and figures generation, QIIME2 artifacts were imported to RStudio (version 2021.09.1 + 372) using qiime2R package (https://github.com/jbisanz/qiime2R; version 0.99.34). Phyloseq (version 1.38.0) [45] objects were produced in RStudio by using “qza_to_phyloseq” function in qiime2R. Dokdo python package (https://github.com/sbslee/dokdo; version 1.6) was used to extract relative abundances of the dominant bacterial phyla from the QIIME2-generated taxa barplots. All figures were generated in RStudio using ggplot2 (version 3.3.5). The RStudio interface (integrated development environment) used for the analyses in this study was based on R version 4.1.2 (R Core Team, 2021).

## Results

### Each test fungus created a predictable wood decay environment

**Fig. 1:**
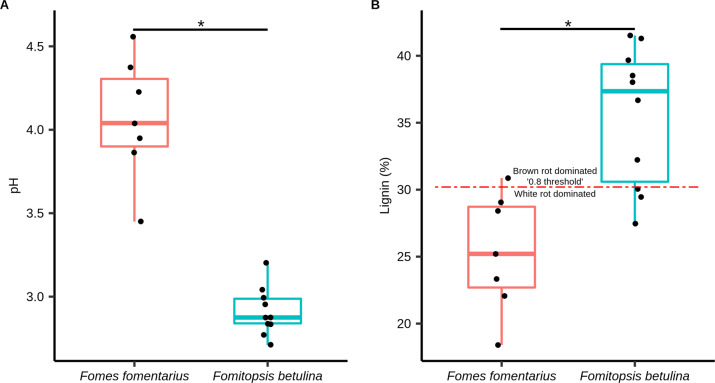
pH and lignin contents (%) of wood samples dominated by *Fomes fomentarius* (white rot) and *Fomitopsis betulina* (brown rot). Box plots represent the pH (**A**) and lignin contents (%) (**B**) of wood samples corresponding to *Fomes fomentarius* and *Fomitopsis betulina*. Statistical significance (*P* < 0.05) is shown by asterisk (*) that was calculated using independent t-test. The red dashed line in **B** represents the “0.8 threshold”, which corresponds to the ratio of lignin loss to density loss (L:D) and delineates brown rot (<0.8) from white rot (>0.8). Samples above the red dashed line in **B** are brown rot-dominated whereas samples below it are white rot-dominated.

Since rot types are known to differentially affect the pH found in deadwood (brown rot pH < white rot pH), in addition to lignin contents [5, 8], we coupled wood pH measurements with lignin measurements. Using samples in which we could confirm the dominance of *F. fomentarius* or *F. betulina* by ITS sequencing, we found the average pH of wood for both rot types were acidic. The brown rot fungus *F. betulina* (average of pH 2.9) was significantly lower (*P* < 0.05) than the white rot fungus *F. fomentarius* (average pH 4.0) (Fig. 1A). All samples representing *F. fomentarius* had a higher pH than the highest pH created by *F. betulina* (Fig. 1A). The average, non-adjusted lignin content for birch samples dominated (relative abundance threshold of 20% based on ITS data; Supplementary Fig. 1) by *F. betulina* sequences (35.5% ± 5.2 standard deviation) was significantly higher than those of *F. fomentarius* (25.3% ± 4.4) and above the 30.2% threshold, confirming that the brown rot-dominated birch had lignin loss patterns in line with brown rot, and vice versa for white rot (Fig. 1B; Supplementary Table 2).

### Bacterial communities associated with each wood rot type were different

The above data suggest that different rot types can alter the microenvironments in deadwood, which could affect the bacterial community associated with each rot type. To determine if there were any significant differences in the bacterial communities associated with each species, we carried out 16S rRNA gene amplicon sequencing for each of the samples we collected. We obtained a total of 258,101 raw reads after demultiplexing the sequencing data from all 27 samples (*F. fomentarius* *n* = 13, *F. betulina* *n* = 12, and two controls samples). After denoising and quality control, we obtained 2039 amplicon sequence variants (ASVs) with a total frequency of 177,513 for all 27 samples (ASVs and “features” are interchangeable). The per-sample minimum and maximum frequencies of ASVs were 1300 and 22,876 with the mean frequency of 6574 (Supplementary Table 3; excluding the blank controls). From this table we filtered all ASVs corresponding to mitochondria, chloroplast and the “decontam-identified” ASV (Feature ID: 5ad0bdb355eee69fed1de03984625e; *Pseudomonas*). To ensure that we were analyzing bacterial communities associated with *F. fomentarius* and *F. betulina*, ITS sequencing of each of the 25 samples collected was used to determine the presence or absence of *F. fomentarius* and *F. betulina*. Of the 25 samples, 17 samples (10 *F. betulina* and 7 *F. fomentarius*) showed enough presence (threshold relative abundance 20% based on ITS data) of the aforementioned fungal species (Supplementary Fig. 1). These eight absences imply that the 10-cm drill depth was in some cases sampling outside of the territories of the fungi inside the wood. Our final dataset for the downstream analyses included a total of 17 samples with 1039 ASVs and a total frequency of 86,548 (Supplementary Table 4). The per-sample minimum and maximum ASV frequencies were 1189 and 9029, respectively, whereas the mean frequency was 5019 (Supplementary Table 4).

**Fig. 2:**
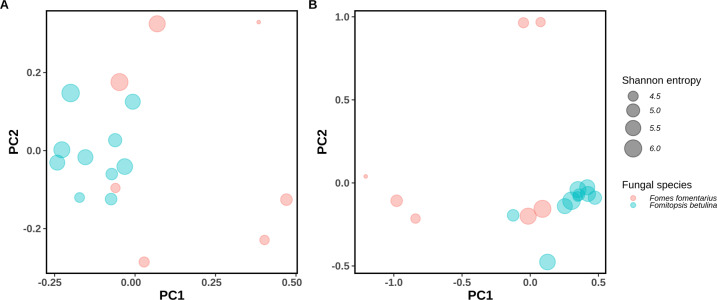
The Principal Coordinate Analysis (PCoA) plot represents beta diversity comparisons of bacterial communities in fungal (*Fomes fomentarius* and *Fomitopsis betulina*) decomposed wood. PCoA of unweighted (**A**) and weighted (**B**) UniFrac distances are shown.

Dead birch trees under the influence of contrasting decomposition mechanisms from *F. betulina* and *F. fomentarius* have comparable levels of bacterial taxa as shown by Shannon entropy (Kruskal-Wallis test; *H* = 2.142, *P* = 0.143), number of observed ASVs [(Kruskal-Wallis test; *H* = 0.955, *P* = 0.328), species richness], Pielou’s evenness (Kruskal-Wallis test; *H* = 1.152, *P* = 0.283) and Faith’s PD (Kruskal-Wallis test; *H* = 0.342, *P* = 0.558) (Supplementary Fig. 2A–D). However, when we compared bacterial profiles associated with each fungal species using beta diversity metrics, we found significant group differences between *F. fomentarius* and *F. betulina* samples. Bacterial communities in the white rot-dominated samples are separated by fungal rot type from the brown rot-dominated communities in beta diversity as measured by weighted UniFrac (PERMANOVA, pseudo-*F* = 3.596, *P* = 0.001, permutations = 999) (Fig. 2A) and unweighted UniFrac (PERMANOVA, pseudo-*F* = 1.826, *P* = 0.001, permutations = 999) distance metrics (Fig. 2B). PCoA plot showed separation by fungal species with samples from *F. fomentarius* scattered on the ordination plot. However, most of the samples (eight out of ten) from brown rot *F. betulina* were tightly clustered in the PCoA plot based on weighted UniFrac distances (Fig. 2B) as compared to unweighted UniFrac distance-based plot (Fig. 2A). The *F. fomentarius* samples clustered to 3 distinct groups, one of which comprising of “*F. fomentarius* 3 and 5” shared close proximity with the *F. betulina* samples (Fig. 2B). Similarly, on the other ordination plot (Fig. 2A), “*F. fomentarius* 3” and “*F. fomentarius* 1” clustered close to *F. betulina* samples. However, neither of the *F. fomentarius* samples had a pH value close to those of the *F. betulina* samples.

### Composition of bacterial communities were distinct in “taxon”-specific ways

**Fig. 3:**
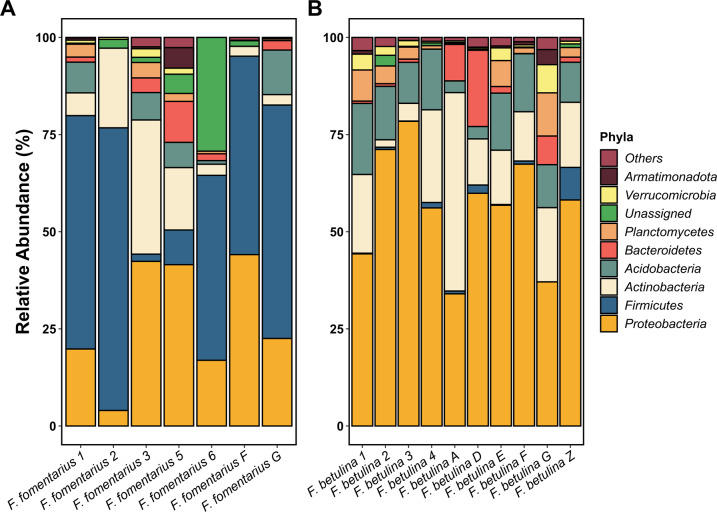
Taxa barplot representing relative abundances (%) of the dominant bacterial phyla associated with *Fomes fomentarius* (white rot) and *Fomitopsis betulina* (brown rot). The percent relative abundances of the most dominant bacterial phyla within each sample of *Fomes fomentarius* (**A**) and *Fomitopsis betulina* (**B**) are shown. “Others” in the figure legend represent a group of bacterial phyla with low percent relative abundances.

**Fig. 4:**
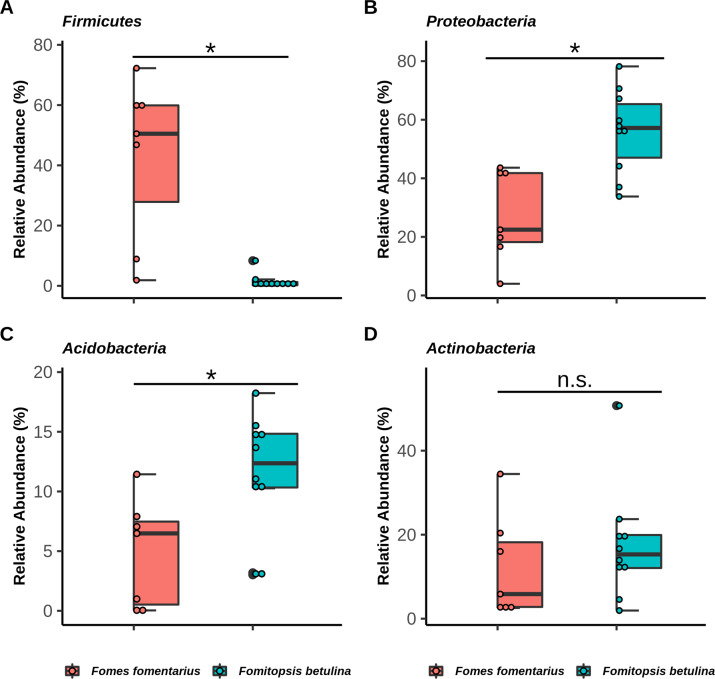
Four dominant bacterial phyla and their percent relative abundances in *Fomes fomentarius* and *Fomitopsis betulina*. **A** *Firmicutes* **B** *Proteobacteria* **C** *Acidobacteria* and **D** *Actinobacteria*. Boxes represent the interquartile range (IQR) and the lines dividing the boxes indicate the median. Statistically significant differences (*P* < 0.05) are represented by an asterisk (*) and calculated using Kruskal-Wallis test.

The dominant phylum across the two fungal-associated communities was *Proteobacteria* followed by *Firmicutes*, *Actinobacteria,* and *Acidobacteria* (Fig. 3). There was also a disproportionate abundance of the phylum *Firmicutes* in the *F. fomentarius* samples, and we found that the difference in relative percent abundance of the ASVs representing *Firmicutes* was statistically significant (Kruskal-Wallis test; *P* value = 0.001) between *F. fomentarius* (mean = 42.8) and *F. betulina* (mean = 1.4) as shown in Fig. 4A, indicating that the *Firmicutes* are enriched in samples with *F. fomentarius. Proteobacteria* and *Acidobacteria* were enriched in the *F. betulina* (mean = 56; mean = 11.5) samples compared to *F. fomentarius* (mean = 27.1; mean = 4.8) (Fig. 3), and this difference was statistically significant (*Proteobacteria*, Kruskal-Wallis test; *P* value = 0.004, and *Acidobacteria*, Kruskal-Wallis test; *P* value = 0.019) (Fig. 4B, C). Among other less dominant phyla, *Actinobacteria* were present in both *F. betulina* (mean = 17.5) and *F. fomentarius* (mean = 12.1) samples without distinct patterns (Fig. 4D; Kruskal-Wallis test; *P* value = 0.435). The remaining phyla including *Bacteroidetes*, *Planctomycetes*, *Verrucomicrobia* and *Armatimonadota* were less abundant across the two fungal groups.

We performed differential-abundance tests using ANCOM-BC and analyzed our data for differentially abundant bacterial taxa between *F. betulina* and *F. fomentarius* groups. We aggregated taxa into six taxonomic levels; Phylum, Class, Order, Family, Genus, and Species. At the phylum level, three phyla including *Firmicutes*, *Proteobacteria* and *Myxococcota* were differentially abundant with *Firmicutes* significantly (Bonferroni-adjusted *P* < 0.05) more abundant in *F. fomentarius* whereas *Proteobacteria* and *Myxococcota* (Bonferroni-adjusted *P* < 0.05) more in *F. betulina* (Supplementary Fig. 3A; Supplementary Table 5). At the class level, *Bacilli* was differentially abundant (Bonferroni-adjusted *P* < 0.05) in *F. fomentarius* along with the *Chthonomonadetes* (Bonferroni-adjusted *P* < 0.05) and *Polyangia* (Bonferroni-adjusted *P* < 0.05), while *Myxococcia* (Bonferroni-adjusted *P* < 0.05) was abundant in *F. betulina* (Supplementary Fig. 3B; Supplementary Table 5). The differential abundance of orders *Paenibacillales* (*Firmicutes*; *Bacilli*) and *Lachnospirales* (*Firmicutes*; *Clostridia*) was significantly higher (Bonferroni-adjusted *P* < 0.05) in *F. fomentarius*. Orders that belonged to *Actinobacteria* (IMCC26256, *Acidimicrobia*_uncultured, and *Bifidobacteriales*) were more abundant (Bonferroni-adjusted *P* < 0.05) in *F. betulina* dominated samples (Supplementary Fig. 3C; Supplementary Table 5).

**Fig. 5:**
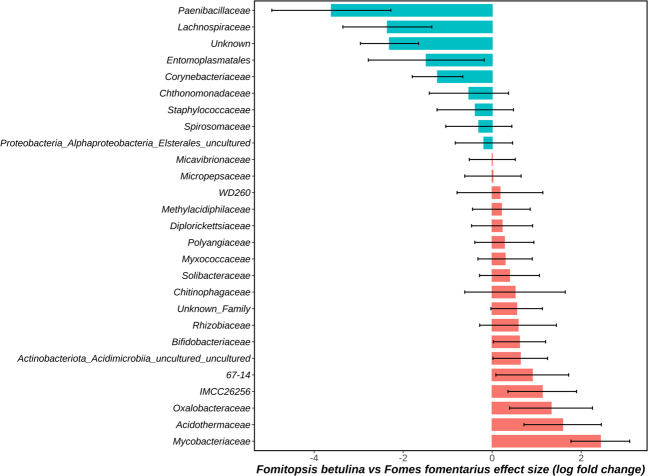
Waterfall plot representing effect size or beta values (i.e., log fold change; *Fomitopsis betulina* versus *Fomes fomentarius*) derived from the ANCOM-BC model. *X*-axis represents log fold change in abundance (differential abundance) of taxa in *Fomitopsis betulina* versus *Fomes fomentarius* whereas *Y*-axis represents differentially abundant taxa at the taxonomic level of Family. All effect sizes with Bonferroni-adjusted *P*  <  0.05 are shown. Differentially abundant bacterial taxa in *Fomes fomentarius* samples are represented by blue bars whereas those represented by red bars are differentially abundant in *Fomitopsis betulina* samples. Supplementary Table 5 includes primary data derived from ANCOM-BC analyses.

The ANCOM-BC analyses of our data revealed significant variations in differential abundances of bacterial families and genera that support our conclusions that *F. fomentarius* tend to correlate preferentially with *Firmicutes* as compared to other bacterial phyla (Fig. 5). For instance, we noticed that *Paenibacillaceae*, *Lachnospiraceae* and *Entomoplasmatales* (*Firmicutes*) were differentially abundant (Bonferroni-adjusted *P* < 0.05) in *F. fomentarius* and were driving the variation between the two groups (*F. betulina* vs *F. fomentarius*). However, several bacterial families such as those belonging to *Actinobacteria* (IMCC26256, *Mycobacteriaceae*, *Acidothermaceae*, 67-14, *Bifidobacteriaceae*) and *Proteobacteria* (*Rhizobiaceae*, *Oxalobacteraceae*) were differentially abundant (Bonferroni-adjusted *P* < 0.05) in *F. betulina*, whereas *Corynebacteriaceae* (*Actinobacteria*) was abundant in *F. fomentarius* (Fig. 5). At the genus level, differential abundances of bacterial taxa between *F. fomentarius* and *F. betulina* followed similar trend as observed for the aforementioned taxonomic levels. *Paenibacillus* and *Spiroplasma* (*Firmicutes*), *Ferruginibacter* (*Bacteroidetes*), *Corynebacterium* (*Actinobacteria*) and *Chthonomonas* (*Armatimonadota*) were abundant in *F. fomentarius* and other genera representing *Acidobacteria* (*Granulicella*, *Bryocella* and *Edaphobacter*), *Actinobacteria* (*Conexibacter*, *Mycobacterium*, *Acidothermus*, IMCC26256, 67-14), *Bacteriodetes* (*Mucilaginibacter*) and *Proteobacteria* were differentially abundant in *F. betulina* samples (Supplementary Fig. 4A; Supplementary Table 5). Overall, it appeared that *F. betulina* associated with a more diverse range of taxa in comparison to *F. fomentarius*, which hosted specific genera and species of *Firmicutes* significantly more than members of other phyla (Supplementary Fig. 4B; Supplementary Table 5).

## Discussion

The influence of factors such as decay stage, season, and tree species on deadwood-dwelling bacterial communities have been studied, but this is the first study to analyze the bacterial communities associated with the two most common and distinct biological routes to decomposing wood (brown and white rot). From a mechanistic point of view, the white rot fungus *F. fomentarius* preferentially decomposes lignin first [18, 19] followed by partial decomposition of the holocellulose [19], whereas the brown rot fungus *F. betulina* is equipped with cellulose- and hemicellulose-degrading enzymes [17] and selectively decomposes the holocellulose portion in wood [19]. Thus, the factors governing their success colonizing wood as well as the fate of the carbon they release will affect carbon dynamics in birch deadwood, and it has larger implications on the role of rot type in controlling carbon released from forests. Most of Earth’s aboveground biomass-sequestered carbon is in wood that will, inevitably, be degraded by these fungi. Bacteria may help shape the dynamics of carbon cycling in forests through their interactions with wood rot fungi, and our results suggest that distinct bacterial communities are associated with fungi that use different mechanisms to decompose wood. We found that there was a significant difference in the relative abundances of particular taxa in bacterial communities associated with either a white rot (*F. fomentarius*) or brown rot (*F. betulina*) fungi.

One of the major findings of the current study was the higher relative abundance of *Firmicutes* in the *F. fomentarius* samples. Although the role of *Firmicutes* in this study is not evident from our analyses, others have recently found that *Firmicutes* play an important role in the fixation of nitrogen and decomposition of biopolymers such as cellulose in deadwood [46]. The performance of these key functions in deadwood microenvironments suggests that *Firmicutes* might not need assistance utilizing sugars and nitrogen in wood [46] and could reflect a complementary physiology with the white rot *F. fomentarius*, which is likely allowing access to embedded carbohydrates. Others have shown an increase in the abundance of *Firmicutes* during the decomposition of macrophyte litters [47] and high level of organic matter [48, 49], suggesting that some members of the *Firmicutes* may compete successfully with other bacteria in carbon-rich environments. *Paenibacillus* was a universal and relatively abundant genus in most *F. fomentarius* samples (Supplementary Fig. 4A, Supplementary Table 5). *Paenibacillus* is an important group of facultative anaerobic bacteria known to encode enzymes involved in the decomposition of lignin [50], and some *Paenibacillus* species can decompose lignin as well as cellulose and hemicellulose [51]. Several species (20 out of 120 species tested) of *Paenibacillus* can fix nitrogen [52], and it is possible that nitrogen-fixing *Paenibacillus* could contribute to nitrogen availability in the otherwise nitrogen-poor environment of deadwood in the early stages of decomposition [46, 53].

We also found that *Acidobacteria* and *Proteobacteria* were significantly more abundant bacterial phyla in *F. betulina* samples as compared to *F. fomentarius* (Fig. 4). With regards to *Acidobacteria*, major genera contributing to significantly different patterns between *F. betulina* and *F. fomentarius* samples were *Granulicella*, *Bryocella* and *Edaphobacter*, both belonging to phylum *Acidobacteria*. *Acidobacteria* prefer acidic environment with higher fluxes of plant-derived organic matter [54, 55] and the genera *Granulicella* and *Edaphobacter* have been shown to encode many carbohydrate-active enzymes and hydrolytic enzymes [56–58]. Recently, genome sequence information of *Granulicella*, *Edaphobacter*, and *Mucilaginibacter* (*Bacteroidetes*) revealed the presence of genes encoding cellulase, hemicellulase, and chitinase enzymes [59]. Unsurprisingly, the pH of *F. betulina* samples was significantly more acidic than that of *F. fomentarius* (Fig. 1). Brown rot fungi reliably produce a much more acidic environment in wood as compared to white rot fungi, a trait that has been well-established in pure culture with no bacteria present [60]. Our results are also in agreement with previous findings for these fungi in decaying birch near these forest sites [8]. The reliable pH distinction between brown and white rot has been linked to higher yields of oxalic acids and lignin phenolics by brown rot fungi [61–65]. Brown rot fungal acidic conditions may enable cellulose degradation via oxalic acid secretion, leading to reduction in extracellular pH and enabling a reactive oxygen Fenton-based mechanism to depolymerize lignocellulose [66]. In Fenton reaction, Fe^3+^ is first mobilized by oxalate anion and then reduced by a quinone to Fe^2+^, which reacts with H_2_O_2_ to produce hydroxyl radicals that oxidatively degrade lignocellulose in wood cell walls [67, 68]. We observed significantly more *Acidobacteria* in *F. betulina* samples, potentially because they tolerate or even thrive in lower pH conditions created by *F. betulina* as part of its Fenton-based mechanism requirement [15]. Notably, pH has been one of the strongest regulators of *Acidobacteria* abundances in soil and most of its members are slow-growing oligotrophs [55, 69].

Our ANCOM-BC analyses also revealed several members of the phylum *Actinobacteria* to be differentially abundant in *F. betulina* samples. *Actinobacteria* and its members have long been associated with decomposing wood and recently *Glaciihabitans* was shown to harbor genes encoding cellulase, alpha-glucanase, and xylobiosidase [59].

Among the *Proteobacteria* associated with the two fungi, a major contribution was from class *Alphaproteobacteria*, which are known for their association with wood rot fungi [46, 53]. *Alphaproteobacteria* such as *Acetobacteraceae*, *Novosphingobium*, *Rhizobium*, *Caulobacteraceae*, *Acidiphilium*, *Methylovirgula,* *Endobacter* and 1174-901-12 (1174-901-12 belongs to the *Beijerinckiaceae*) were differentially present in *F. betulina* compared to *F. fomentarius* samples. The presence of these bacterial groups in deadwood is not surprising as they were previously reported in similar environments [16, 53]. Members of *Beijerinckiaceae* are known for their ability to carry out methanotrophy, methylotrophy and nitrogen fixation in acidic environments [70, 71]. Recently it was shown that *Proteobacteria* in deadwood are associated with methylotrophy [46] and utilize low-molecular-weight carbon sources and can access carbon through mycophagy [59]. *Acidiphilium* (*Acetobacteraceae*) is an extreme acidophile and most of its members are heterotrophs except for the facultative autotroph *Acidiphilium acidophilum* that utilizes sulfur (zero-valent) for fueling carbon dioxide fixation [72]. *Endobacter* belongs to *Acetobacteraceae* and can grow at extreme acidic pH and exist as endophytes in the nodules of legume plants [73]. The presence of *Endobacter* and other members of *Acetobacteraceae* associated with *F. betulina* may therefore reflect the lower pH in *F. betulina* samples.

Here we describe, to our knowledge, the first study that used two fungi with contrasting wood decomposition mechanisms and highlighted their associations with bacterial communities. Our results provide a solid foundation for future studies, but also highlight the need for approaches such as metagenome-assembled genomes and metatranscriptomics to give insight into the functional capabilities of the associated bacterial communities. Although we show that fungal wood decay mechanisms seem to be associated with distinct bacterial community composition, more work is needed to determine if these findings are applicable to other white and brown rot fungi. We also need to study whether the fungal environment promotes certain bacterial associations, or if, by contrast, the bacteria somehow promote one rot type over another. In either case, if these distinct fungi–bacteria relationships shape the viability of one rot type relative to another, with such large implications on woody carbon, we need to include bacteria among the biotic factors that might improve predictions of wood decay, carbon cycling, and climate change.

## Supplementary information

Supplementary Figures

Supplementary Tables

## Data Availability

Both bacterial and fungal raw sequence data were deposited to the Sequence Read Archive (SRA) of the National Center of Biotechnology Information (NCBI) under the BioProject number PRJNA752646. All codes and parameters pertaining to the analyses in this study are publicly available at https://github.com/IrshadUlHaq1/parameters.
