# Supplementary material for: Bacterial communities associated with wood rot fungi that use distinct decomposition mechanisms
Source: ISME Commun. 2022 Mar 30;2:26. doi: 10.1038/s43705-022-00108-5 (PMC9723729; doi:10.1038/s43705-022-00108-5)
Supplement: Supplementary file 1 — Supplementary Figures [file 43705_2022_108_MOESM1_ESM.pdf]

Supplementary Information

Bacterial communities associated with wood rot fungi that use distinct decomposition mechanisms

Irshad Ul Haq[1,4], Benjamin Hillmann[2], Molly Moran[1], Samuel Willard[3], Dan Knights[2,4], Kathryn R. Fixen[1,4], Jonathan S. Schilling[1,4,a]

[1]Department of Plant and Microbial Biology, College of Biological Sciences, University of Minnesota, St. Paul, MN, United States

[2]Department of Computer Science and Engineering, University of Minnesota, Minneapolis, MN, United States

[3]Department of Life Sciences, Imperial College London, London, UK

[4]Biotechnology Institute, College of Biological Sciences, University of Minnesota, Minneapolis, MN, United States

[a]

Corresponding address: schillin@umn.edu

Running title: Microbial communities associated with wood rot fungi

This file includes **supplementary Figure 1**, **supplementary Figure 2, supplementary Figure 3 and supplementary Figure 4**

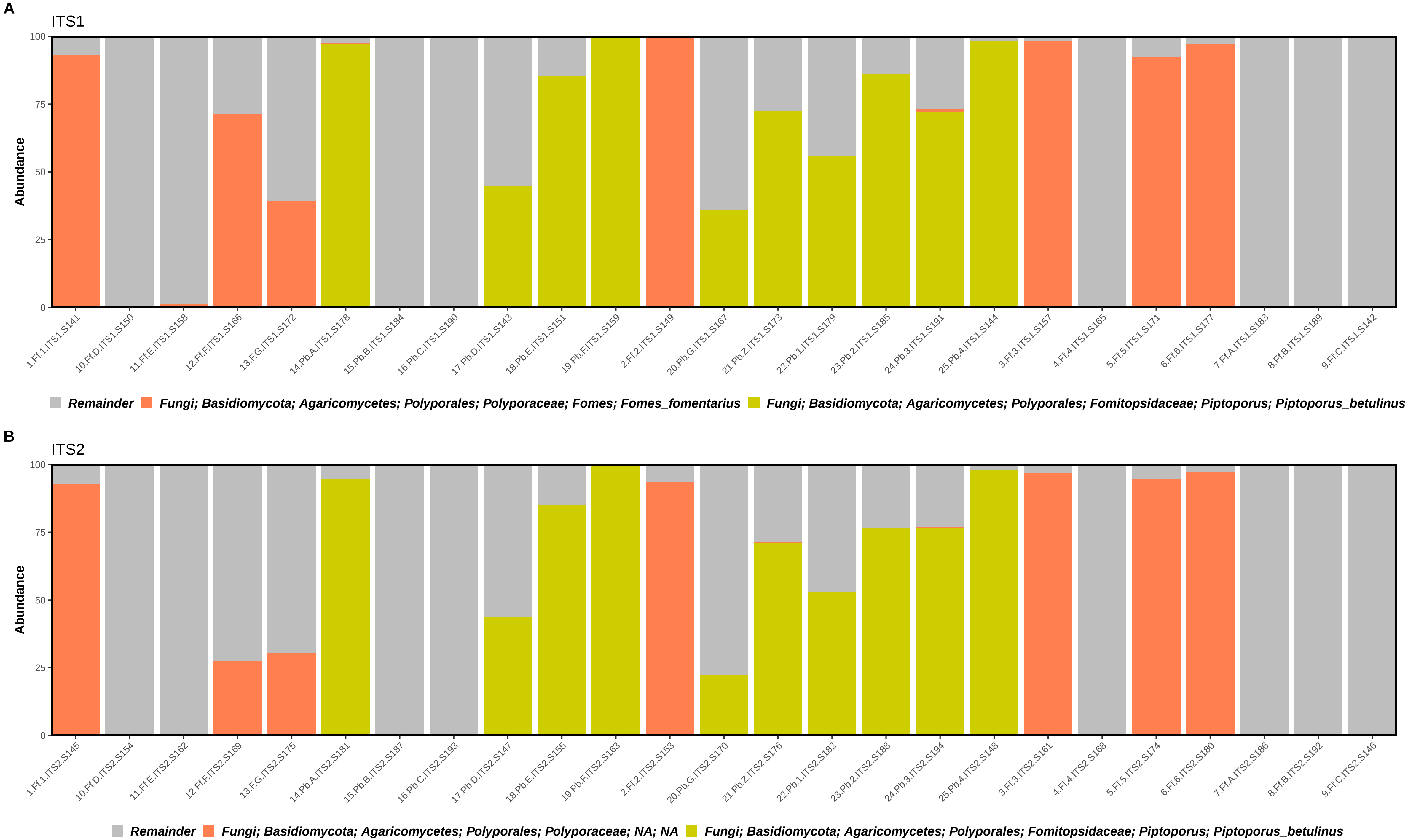

Supplementary Figure 1: Taxa bar plots representing the percent relative abundances of Fomes fomentarius, Fomitopsis betulina and the remainder fungal species in samples collected from decomposed standing birch trees. A and B represents ITS1 and ITS2 data, respectively.

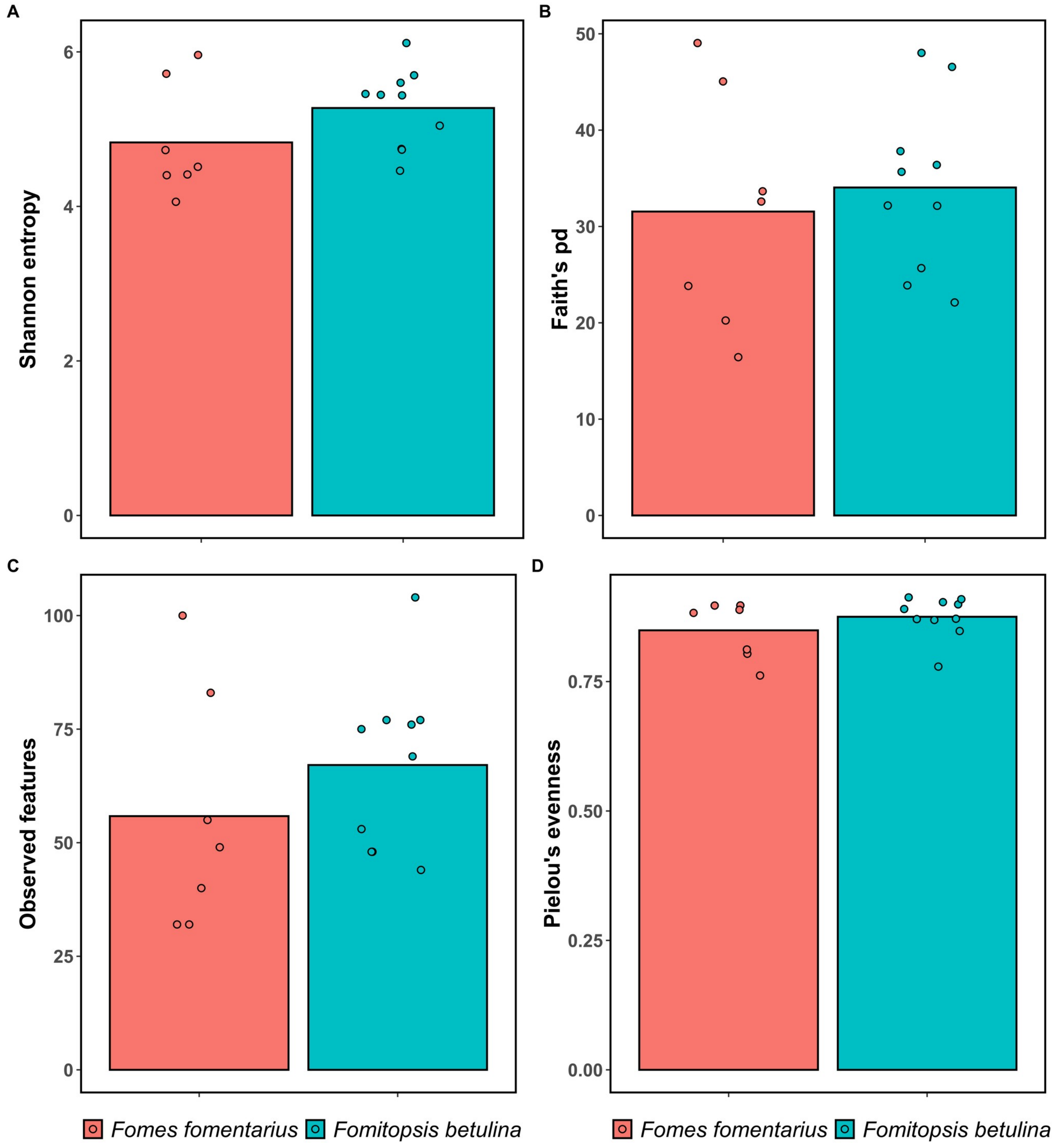

Supplementary Figure 2: Alpha diversity measures of Shannon entropy **(A)** Faith phylogenetic distance **(B)** observed features/species richness **(C)** and Pielou's evenness **(D)** assessed with a nonparametric Kruskal-Wallis test and Benjamini and Hochberg correction. None of the differences were statistically significant.

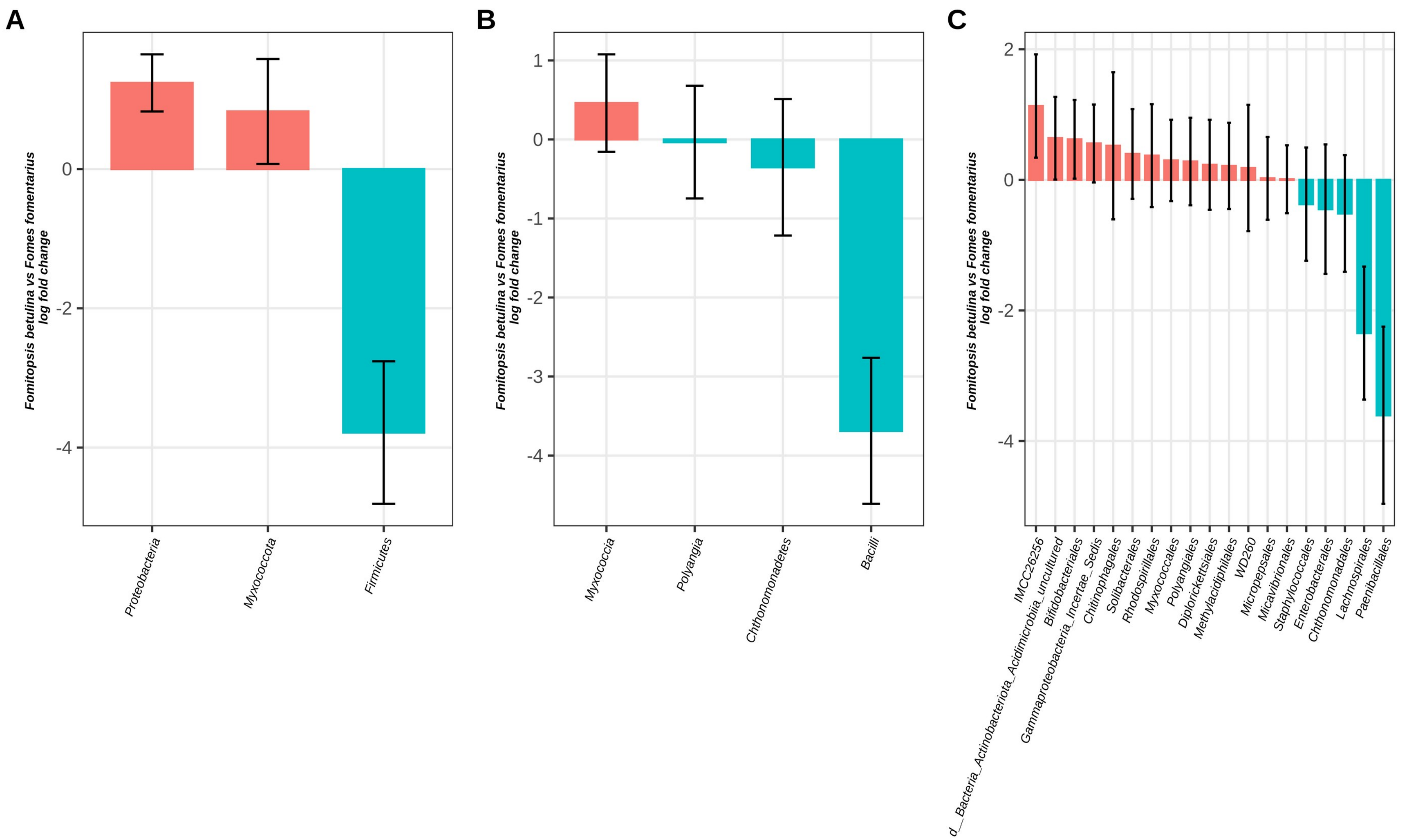

Supplementary Figure 3. Waterfall plot representing effect size or beta values (log fold change; *F. betulina* versus *F. fomentarius*) derived from the ANCOM-BC model. X-axis shows bacterial taxa while Y-axis represents log fold change in abundance of taxa in *F. betulina* versus *F. fomentarius*. Taxa represented by blue bars are abundant in *F. fomentarius* whereas those represented by red bars are abundant in *F. betulina*. Exact effect size or beta values, W values and adjusted *P* values are provided in Supplementary Table 5. Taxa at the level of Phylum **(A)**, Class **(B)** and Order **(C)** are shown.

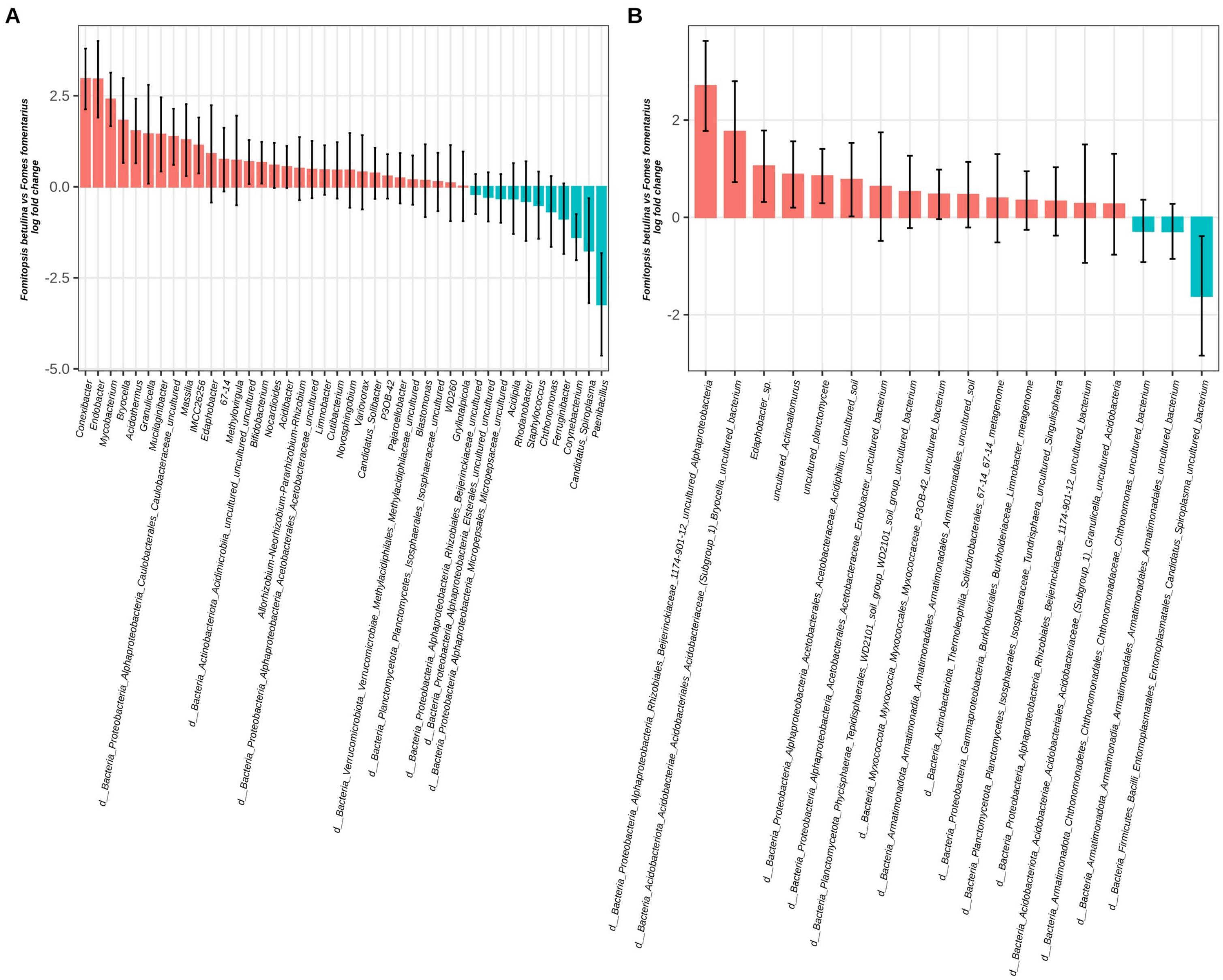

Supplementary Figure 4. Waterfall plot representing effect size or beta values (log fold change; *F. betulina* versus *F. fomentarius*) derived from the ANCOM-BC model. X-axis shows bacterial taxa while Y-axis represents log fold change in abundance of taxa in *F. betulina* versus *F. fomentarius*. Taxa represented by blue bars are abundant in *F. fomentarius* whereas those represented by red bars are abundant in *F. betulina*. Exact effect size or beta values, W values and adjusted *P* values are provided in Supplementary Table 5. Taxa at the level of Genus **(A)** and Species **(B)** are shown.
