# Supplementary material for: Bacterial communities associated with wood rot fungi that use distinct decomposition mechanisms
Source: ISME Commun. 2022 Mar 30;2:26. doi: 10.1038/s43705-022-00108-5 (PMC9723729; doi:10.1038/s43705-022-00108-5)
Supplement: Supplementary file 2 — Supplementary Tables [file 43705_2022_108_MOESM2_ESM.pdf]

Supplementary Information

Bacterial communities associated with wood rot fungi that use distinct decomposition mechanisms

Irshad Ul Haq[1,4], Benjamin Hillmann[2], Molly Moran[1], Samuel Willard[3], Dan Knights[2,4], Kathryn R. Fixen[1,4], Jonathan S. Schilling[1,4,a]

[1]Department of Plant and Microbial Biology, College of Biological Sciences, University of Minnesota, St. Paul, MN, United States

[2]Department of Computer Science and Engineering, University of Minnesota, Minneapolis, MN, United States

[3]Department of Life Sciences, Imperial College London, London, UK

[4]Biotechnology Institute, College of Biological Sciences, University of Minnesota, Minneapolis, MN, United States

[a]**Corresponding address**: schillin@umn.edu

Running title: Microbial communities associated with wood rot fungi

This supplementary information file includes supplementary table 1, supplementary table 2, supplementary table 3, supplementary table 4 and supplementary table 5.

Supplementary Table 1: List of samples, names key and samples' collection dates

Sample name	Birch tree	Collection Date	Sample name	Birch tree	Collection Date
Fomitopsis betulina 1	Tree #1	12/19/18	*Fomes fomentarius* 1	Tree #1	12/19/18
Fomitopsis betulina 2	Tree #2	12/19/18	*Fomes fomentarius* 2	Tree #2	12/19/18
Fomitopsis betulina A	Tree #3	01/17/19	*Fomes fomentarius* A	Tree #3	01/17/19
Fomitopsis betulina B	Tree #4	01/17/19	*Fomes fomentarius* B	Tree #4	01/17/19
Fomitopsis betulina C	Tree #5	01/17/19	*Fomes fomentarius* C	Tree #5	01/17/19
Fomitopsis betulina D	Tree #6	01/17/19	*Fomes fomentarius* D	Tree #6	01/17/19
Fomitopsis betulina E	Tree #7	01/17/19	*Fomes fomentarius* E	Tree #7	01/17/19
Fomitopsis betulina F	Tree #8	01/17/19	*Fomes fomentarius* F	Tree #8	01/17/19
Fomitopsis betulina G	Tree #9	01/17/19	*Fomes fomentarius* G	Tree #9	01/17/19
Fomitopsis betulina Z	Tree #10	01/17/19	*Fomes fomentarius* 3	Tree #13	12/19/18
Fomitopsis betulina 3	Tree #11	12/19/18	*Fomes fomentarius* 4	Tree #14	12/19/18
Fomitopsis betulina 4	Tree #12	12/19/18	*Fomes fomentarius* 5	Tree #15	12/19/18
			Fomes fomentarius 6	Tree #16	12/19/18

Birch trees with both *Fomes fomentarius* and *Fomitopsis betulina*

Birch trees with either *Fomes fomentarius* or *Fomitopsis betulina*

Samples included in the final analyses (*Fomes fomentarius*; n=07, *Fomitopsis betulina*; n=10)

Trees with both *Fomes fomentarius* and *Fomitopsis betulina* = 09

Trees with only *Fomes fomentarius* = 04

Trees with only *Fomitopsis betulina* = 03

Total trees = 16

Supplementary Table 2: Lists of samples with their respective lignin contents

Sample ID	Sample name	Mass (g)	Boat (g)	Filter (g)	OD Total	Acid-insoluble lignin (g)	Lignin %	Average % Lignin	Standard deviation
Ff.1	*Fomes fomentarius* 1	0.0603	2.5431	0.1346	2.6888	0.0111	18.4079601990055	25.3332246099975	4.40900223699111
Ff.2	*Fomes fomentarius* 2	0.06	2.4938	0.1347	2.6425	0.014	23.3333333333337		
Ff.3	*Fomes fomentarius* 3	0.0602	2.4662	0.1346	2.6179	0.0171	28.4053156146181		
Ff.5	*Fomes fomentarius* 5	0.0599	2.5194	0.1342	2.6687	0.0151	25.2086811352252		
Ff.6	*Fomes fomentarius* 6	0.0603	2.524	0.1343	2.6716	0.0133	22.0563847429521		
Ff.F	*Fomes fomentarius* F	0.0596	2.5356	0.1351	2.6891	0.0184	30.8724832214761		
Ff.G	*Fomes fomentarius* G	0.0599	2.5276	0.134	2.679	0.0174	29.0484140233721		
Pb. 1	*Fomitopsis betulina* 1	0.0605	2.5386	0.1334	2.6953	0.0233	38.5123966942147	35.4874398291659	5.22153066414595
Pb. 2	*Fomitopsis betulina* 2	0.0601	2.4825	0.1349	2.6351	0.0177	29.450915141431		
Pb. 3	*Fomitopsis betulina* 3	0.0595	2.5151	0.1368	2.6766	0.0247	41.5126050420171		
Pb. 4	*Fomitopsis betulina* 4	0.0599	2.522	0.134	2.6753	0.0193	32.2203672787985		
Pb. A	*Fomitopsis betulina* A	0.0599	2.5365	0.134	2.6885	0.018	30.0500834724537		
Pb. D	*Fomitopsis betulina* D	0.0595	2.5189	0.1351	2.6776	0.0236	39.6638655462186		
Pb. E	*Fomitopsis betulina* E	0.0605	2.5238	0.1353	2.6821	0.023	38.01652892562		
Pb. F	*Fomitopsis betulina* F	0.0603	2.4863	0.1351	2.6463	0.0249	41.2935323383087		
Pb. G	*Fomitopsis betulina* G	0.0597	2.4134	0.1336	2.5634	0.0164	27.4706867671691		
Pb. Z	*Fomitopsis betulina* Z	0.0597	2.5107	0.1344	2.667	0.0219	36.6834170854272		
C (day 1)	C (day 1)	0.0603	2.538	0.1349	2.6855	0.0126	20.8955223880603		
C (day 2)	C (day 2)	0.0599	2.5401	0.1345	2.689	0.0144	24.0400667779636		

Supplementary Table 3: Detail of feature frequencies of all samples

Sample Identifiers	Sample names	Frequency per sample
8.Ff.B.16S.S138.001	*Fomes fomentarius* B	22876
7.Ff.A.16S.S135.001	*Fomes fomentarius* A	16103
16.Pb.C.16S.S139.001	*Fomitopsis betulina* C	12068
20.Pb.G.16S.S128.001	*Fomitopsis betulina* G	10154
1.Ff.1.16S.S114.001	*Fomes fomentarius* 1	9561
24.Pb.3.16S.S140.001	*Fomitopsis betulina* 3	9532
17.Pb.D.16S.S116.001	*Fomitopsis betulina* D	8898
3.Ff.3.16S.S122.001	*Fomes fomentarius* 3	8083
19.Pb.F.16S.S124.001	*Fomitopsis betulina* F	7916
13.F.G.16S.S130.001	*Fomes fomentarius* G	7125
22.Pb.1.16S.S134.001	*Fomitopsis betulina* 1	6567
11.Ff.E.16S.S123.001	*Fomes fomentarius* E	6347
18.Pb.E.16S.S120.001	*Fomitopsis betulina* E	6278
15.Pb.B.16S.S136.001	*Fomitopsis betulina* B	5742
9.Ff.C.16S.S115.001	*Fomes fomentarius* C	5331
5.Ff.5.16S.S129.001	*Fomes fomentarius* 5	4861
10.Ff.D.16S.S119.001	*Fomes fomentarius* D	4452
23.Pb.2.16S.S137.001	*Fomitopsis betulina* 2	4315
4.Ff.4.16S.S126.001	*Fomes fomentarius* 4	4115
21.Pb.Z.16S.S131.001	*Fomitopsis betulina* Z	3960
2.Ff.2.16S.S118.001	*Fomes fomentarius* 2	3427
14.Pb.A.16S.S133.001	*Fomitopsis betulina* A	3004
25.Pb.4.16S.S117.001	*Fomitopsis betulina* 4	2979
12.Ff.F.16S.S127.001	*Fomes fomentarius* F	2099
6.Ff.6.16S.S132.001	*Fomes fomentarius* 6	1300
BLANK.001.C04.16S.S125.001	Blank (control)	254
BLANK.001.B04.16S.S121.001	Blank (control)	166
**Number of samples**	27	
**Number of features**	2039	
**Total frequency**	177513	
**Maximum frequency per sample**	22876	
**Minimum frequency per sample**	166	

Supplementary Table 4: Detail of feature frequencies of 17 samples

Sample Identifiers	Sample names	Frequency per sample
1.Ff.1.16S.S114.001	*Fomes fomentarius* 1	9029
20.Pb.G.16S.S128.001	*Fomitopsis betulina* G	8816
17.Pb.D.16S.S116.001	*Fomitopsis betulina* D	7886
24.Pb.3.16S.S140.001	*Fomitopsis betulina* 3	7419
19.Pb.F.16S.S124.001	*Fomes fomentarius* 3	7114
3.Ff.3.16S.S122.001	*Fomitopsis betulina* F	7114
13.F.G.16S.S130.001	*Fomes fomentarius* G	6343
22.Pb.1.16S.S134.001	*Fomitopsis betulina* 1	6147
18.Pb.E.16S.S120.001	*Fomitopsis betulina* E	5073
5.Ff.5.16S.S129.001	*Fomes fomentarius* 5	4203
21.Pb.Z.16S.S131.001	*Fomitopsis betulina* Z	3115
2.Ff.2.16S.S118.001	*Fomes fomentarius* 2	3005
14.Pb.A.16S.S133.001	*Fomitopsis betulina* A	2896
23.Pb.2.16S.S137.001	*Fomitopsis betulina* 2	2764
25.Pb.4.16S.S117.001	*Fomitopsis betulina* 4	2653
12.Ff.F.16S.S127.001	*Fomes fomentarius* F	1782
6.Ff.6.16S.S132.001	*Fomes fomentarius* 6	1189
**Number of samples**	17	
**Number of features**	1039	
**Total frequency**	86548	
**Maximum frequency per sample**	9029	
**Minimum frequency per sample**	1189	

Supplementary Table 5: ANCOM-BC data corresponding to differentially abundant taxa between *Fomitopsis betulina* and *Fomes fomentarius*

Taxonomic level	taxa	beta	se	W	p_val	q_val	diff_abn
**Phylum**	*Firmicutes*	-3.78	1.02	-3.7	2.19E-04	0	TRUE
	Myxococcota	0.83	0.75	1.1	0	0	TRUE
	Proteobacteria	1.24	0.41	3.01	0	0.04	TRUE
**Class**	*Chthonomonadetes*	-0.35	0.86	-0.41	0	0	TRUE
	Bacilli	-3.69	0.92	-3.99	6.60E-05	0	TRUE
	Myxococcia	0.46	0.62	0.74	0	0	TRUE
	Polyangia	-0.03	0.71	-0.05	0	0	TRUE
**Order**	*Solibacterales*	0.4	0.69	0.58	0	0	TRUE
	IMCC26256	1.13	0.79	1.43	0	0	TRUE
	Actinobacteriota_Acidimicrobiia_uncultured	0.64	0.63	1.01	0	0	TRUE
	Bifidobacteriales	0.62	0.6	1.03	0	0	TRUE
	Chthonomonadales	-0.51	0.89	-0.58	0	0	TRUE
	Chitinophagales	0.52	1.13	0.46	0	0	TRUE
	Paenibacillales	-3.61	1.36	-2.66	0	0	TRUE
	Staphylococcales	-0.37	0.87	-0.43	0	0	TRUE
	Lachnospirales	-2.35	1.02	-2.3	0	0	TRUE
	Myxococcales	0.3	0.62	0.48	0	0	TRUE
	Polyangiales	0.28	0.67	0.42	0	0	TRUE
	Micavibrionales	0.01	0.52	0.02	0	0	TRUE
	Micropepsales	0.02	0.63	0.04	0	0	TRUE
	Rhodospirillales	0.37	0.79	0.47	0	0	TRUE
	Diplorickettsiales	0.23	0.69	0.33	0	0	TRUE
	Enterobacterales	-0.45	0.99	-0.45	0	0	TRUE
	Gammaproteobacteria_Incertae_Sedis	0.56	0.6	0.93	0	0	TRUE
	WD260	0.18	0.97	0.19	0	0	TRUE

Taxonomic level	taxa	beta	se	W	p_val	q_val	diff_abn
Family	*Solibacteraceae*	0.39	0.67	0.58	0	0	TRUE
	IMCC26256	1.12	0.77	1.46	0	0	TRUE
	Actinobacteriota_Acidimicrobiia_uncultured_uncultured	0.63	0.61	1.03	0	0	TRUE
	Bifidobacteriaceae	0.61	0.59	1.04	0	0	TRUE
	Corynebacteriaceae	-1.23	0.57	-2.16	0	0	TRUE
	Mycobacteriaceae	2.43	0.66	3.69	0	0	TRUE
	Acidothermaceae	1.58	0.87	1.82	0	0	TRUE
	67-14	0.9	0.82	1.1	0	0	TRUE
	Chthonomonadaceae	-0.52	0.89	-0.59	0	0	TRUE
	Chitinophagaceae	0.51	1.13	0.46	0	0	TRUE
	Spirosomaceae	-0.3	0.74	-0.4	0	0	TRUE
	Entomoplasmatales	-1.48	1.3	-1.14	0	0	TRUE
	Paenibacillaceae	-3.61	1.34	-2.7	0	0	TRUE
	Staphylococcaceae	-0.38	0.86	-0.44	0	0	TRUE
	Lachnospiraceae	-2.35	1	-2.36	0	0	TRUE
	Myxococcaceae	0.29	0.61	0.48	0	0	TRUE
	Polyangiaceae	0.27	0.66	0.41	0	0	TRUE
	Proteobacteria_Alphaproteobacteria_Elsterales_uncultured	-0.18	0.64	-0.29	0	0	TRUE
	Micavibrionaceae	0	0.52	0.01	0	0	TRUE
	Micropepsaceae	0.02	0.63	0.03	0	0	TRUE
	Rhizobiaceae	0.58	0.86	0.68	0	0	TRUE
	Oxalobacteraceae	1.32	0.93	1.42	0	0	TRUE
	Diplorickettsiaceae	0.22	0.69	0.33	0	0	TRUE
	Unknown_Family	0.55	0.58	0.95	0	0	TRUE
	WD260	0.18	0.96	0.18	0	0	TRUE
	Methylacidiphilaceae	0.21	0.65	0.32	0	0	TRUE
	Unknown	-2.31	0.65	-3.53	4.09E-04	0.03	TRUE
Genus	*Acidipila*	-0.32	0.97	-0.33	0	0	TRUE
	Bryocella	1.82	1.16	1.56	0	0	TRUE

	Edaphobacter	0.9	1.34	0.68	0	0	TRUE
	Granulicella	1.44	1.36	1.06	0	0	TRUE
	Candidatus_Solibacter	0.37	0.7	0.53	0	0	TRUE
	IMCC26256	1.13	0.77	1.48	0	0	TRUE
	Actinobacteriota_Acidimicrobiia_uncultured_uncultured_uncultured	0.68	0.61	1.12	0	0	TRUE
	Bifidobacterium	0.66	0.57	1.15	0	0	TRUE
	Corynebacterium	-1.38	0.63	-2.18	0	0	TRUE
	Mycobacterium	2.4	0.73	3.27	0	0	TRUE
	Acidothermus	1.53	0.89	1.73	0	0	TRUE
	Gryllotalpicola	0.01	0.96	0.01	0	0	TRUE
	Nocardioides	0.59	0.62	0.95	0	0	TRUE
	Cutibacterium	0.45	0.77	0.58	0	0	TRUE
	67-14	0.75	0.87	0.85	0	0	TRUE
	Conexibacter	2.96	0.83	3.56	0	0	TRUE
	Chthonomonas	-0.68	0.97	-0.7	0	0	TRUE
	Ferruginibacter	-0.88	0.97	-0.91	0	0	TRUE
	Mucilaginibacter	1.44	1.02	1.41	0	0	TRUE
	Candidatus_Spiroplasma	-1.75	1.44	-1.22	0	0	TRUE
	Paenibacillus	-3.23	1.41	-2.29	0	0	TRUE
	Staphylococcus	-0.5	0.92	-0.55	0	0	TRUE
	P3OB-42	0.29	0.61	0.47	0	0	TRUE
	Pajaroellobacter	0.23	0.69	0.34	0	0	TRUE
	Planctomycetota_Planctomycetes_Isosphaerales_Isosphaeraceae_uncultured	0.13	0.8	0.17	0	0	TRUE
	Endobacter	2.95	1.05	2.81	0	0	TRUE
	Proteobacteria_Alphaproteobacteria_Acetobacterales_Acetobacteraceae_uncultured	0.47	0.79	0.6	0	0	TRUE
	Proteobacteria_Alphaproteobacteria_Caulobacterales_Caulobacteraceae_uncultured	1.37	0.77	1.78	0	0	TRUE
	Proteobacteria_Alphaproteobacteria_Elsterales_uncultured_uncultured	-0.28	0.68	-0.41	0	0	TRUE
	Proteobacteria_Alphaproteobacteria_Micropepsales_Micropepsaceae_uncultured	-0.32	0.67	-0.48	0	0	TRUE
	Methylovirgula	0.72	1.23	0.59	0	0	TRUE
	Proteobacteria_Alphaproteobacteria_Rhizobiales_Beijerinckiaceae_uncultured	-0.2	0.55	-0.36	0	0	TRUE

	Allorhizobium-Neorhizobium-Pararhizobium-Rhizobium	0.5	0.86	0.58	0	0	TRUE
	Blastomonas	0.17	1	0.17	0	0	TRUE
	Novosphingobium	0.45	1.02	0.44	0	0	TRUE
	Limnobacter	0.46	0.68	0.67	0	0	TRUE
	Variovorax	0.4	1.02	0.39	0	0	TRUE
	Massilia	1.28	0.99	1.3	0	0	TRUE
	Acidibacter	0.54	0.58	0.94	0	0	TRUE
	WD260	0.1	1.04	0.1	0	0	TRUE
	Rhodanobacter	-0.39	1.09	-0.36	0	0	TRUE
	Verrucomicrobiota_Verrucomicrobiae_Methylacidiphilales_Methylacidiphilaceae_uncultured	0.18	0.68	0.27	0	0	TRUE
**Species**	*Acidobacteriota_Acidobacteriae_Acidobacteriales_Acidobacteriaceae_(Subgroup_1)_Bryocella_uncultured_bacterium*	1.76	1.04	1.7	0	0	TRUE
	Edaphobacter_sp.	1.05	0.73	1.43	0	0	TRUE
	Acidobacteriota_Acidobacteriae_Acidobacteriales_Acidobacteriaceae_(Subgroup_1)_Granulicella_uncultured_Acidobacteria	0.27	1.04	0.26	0	0	TRUE
	uncultured_Actinoallomurus	0.88	0.68	1.29	0	0	TRUE
	Actinobacteriota_Thermoleophilia_Solirubrobacterales_67-14_67-14_metagenome	0.39	0.91	0.43	0	0	TRUE
	Armatimonadota_Armatimonadia_Armatimonadales_Armatimonadales_Armatimonadales_uncultured_bacterium	-0.29	0.57	-0.51	0	0	TRUE
	Armatimonadota_Armatimonadia_Armatimonadales_Armatimonadales_Armatimonadales_uncultured_soil	0.47	0.67	0.69	0	0	TRUE
	Armatimonadota_Chthonomonadetes_Chthonomonadales_Chthonomonadaceae_Chthonomonas_uncultured_bacterium	-0.28	0.64	-0.43	0	0	TRUE
	Firmicutes_Bacilli_Entomoplasmatales_Entomoplasmatales_Candidatus_Spiroplasma_uncultured_bacterium	-1.61	1.23	-1.31	0	0	TRUE
	Myxococcota_Myxococcia_Myxococcales_Myxococcaceae_P3OB-42_uncultured_bacterium	0.47	0.51	0.93	0	0	TRUE
	Planctomycetota_Phycisphaerae_Tepidisphaerales_WD2101_soil_group_WD2101_soil_group_uncultured_bacterium	0.52	0.74	0.7	0	0	TRUE
	uncultured_planctomycete	0.85	0.56	1.52	0	0	TRUE
	Planctomycetota_Planctomycetes_Isosphaerales_Isosphaeraceae_Tundrisphaera_uncultured_Singulisphaera	0.33	0.7	0.47	0	0	TRUE
	Proteobacteria_Alphaproteobacteria_Acetobacterales_Acetobacteraceae_Acidiphilium_uncultured_soil	0.77	0.76	1.03	0	0	TRUE
	Proteobacteria_Alphaproteobacteria_Acetobacterales_Acetobacteraceae_Endobacter_uncultured_bacterium	0.63	1.12	0.57	0	0	TRUE

	Proteobacteria_Alphaproteobacteria_Rhizobiales_Beijerinckiaceae_1174-901-12_uncultured_Alphaproteobacteria	2.7	0.93	2.92	0	0	TRUE
	Proteobacteria_Alphaproteobacteria_Rhizobiales_Beijerinckiaceae_1174-901-12_uncultured_bacterium	0.28	1.22	0.23	0	0	TRUE
	Proteobacteria_Gammaproteobacteria_Burkholderiales_Burkholderiaceae_Limnobacter_metagenome	0.35	0.6	0.58	0	0	TRUE
